# Tumor necrosis factor-α-inducible protein 8-like protein 3 (TIPE3): a novel prognostic factor in colorectal cancer

**DOI:** 10.1186/s12885-023-10590-2

**Published:** 2023-02-08

**Authors:** Yue Xu, Yong Zhu, Hengbo Xia, Yanan Wang, Lin Li, Hong Wan, Shuping Zhang, Aman Xu, Liecheng Wang, Jiao Gong, Pingping Zhang

**Affiliations:** 1 Department of Physiology, School of Basic Medical Sciences, Anhui Medical University, Hefei, China; 2 Department of General Surgery, The First Affiliated Hospital of Anhui Medical University, Hefei, China; 3 Anhui Public Health Clinical Center, Hefei, China; 4 Department of Laboratory Medicine, Third Affiliated Hospital of Sun Yat-sen University, Guangzhou, China

**Keywords:** TIPE3, Colorectal cancer, Immune infiltration, Prognosis

## Abstract

**Background:**

To explore the correlation of tumor necrosis factor-α-induced protein 8-like protein 3 (TIPE3) expressions in colorectal cancer (CRC) with tumor-immune infiltration and patient prognosis.

**Methods:**

Formalin-fixed paraffin-embedded tumor samples from CRC patients (*n* = 110) were used in this study. Immunohistochemistry staining of TIPE3 and three prognostic immune biomarkers (CD8, CD20, and CD66b) was conducted in the tumor tissues and adjacent normal tissues. A Cox regression analysis of univariate and multivariate variables was performed to assess the correlation between TIPE3 and patient prognosis.

**Result:**

We found that TIPE3 was mainly expressed in the cytoplasm, with a small amount in the nucleus. The expression of TIPE3 in tumor tissues is significantly higher than in adjacent normal tissues, and it is significantly correlated with the survival rate of patients in tumor tissues (*p* = 0.0038) and adjacent normal tissues (*p*<0.0001). Patients with a high TIPE3 expression had a lower survival rate, while patients with a low TIPE3 expression had a higher survival rate. Univariate regression analysis showed that the TIPE3 expression in tumor tissues (*p* = 0.007), the TIPE3 expression in adjacent normal tissues (*p*<0.001), the number of CD8+ T cells in tumor tissues (*p* = 0.020), the number of CD20+ B cells in tumor tissues (*p* = 0.023), the number of CD20+ B cells in adjacent normal tissues (*p* = 0.023), the number of CD66b+ neutrophils in tumor tissues (*p* = 0.005), the number of CD66b+ neutrophils in adjacent normal tissues (*p*<0.001), lymphatic metastasis (*p* = 0.010), TNM stage (*p* = 0.013), and tumor grade (*p* = 0.027) were significantly correlated with overall survival (OS). These prognostic factors were then subjected to multivariate regression analysis, and the results showed that the expression of TIPE3, the number of CD8+ T cells, and the number of CD66b+ neutrophils were prognostic factors affecting the OS rate of CRC patients.

**Conclusion:**

We found that the TIPE3 protein is upregulated in CRC cancer tissues and is correlated with survival rate.

**Supplementary Information:**

The online version contains supplementary material available at 10.1186/s12885-023-10590-2.

## Introduction

Colorectal cancer (CRC) is the most common malignant tumor of the digestive system and is associated with a high mortality rate [1, 2]. The occurrence and development of CRC are closely related to patients’ age, gender, lifestyle, dietary habits, and genetic factors [3].

The tumor necrosis factor-α-induced protein 8 family (TNFAIP8/TIPE) is induced by tumor necrosis factor-α (TNF-α), which is closely related to immune regulation and tumorigenesis [4]. It has been reported that this family contains four members, namely, TNFAIP8/TIPE, TIPE1, TIPE2, and TIPE3 [5]. TIPE is a negative regulator of apoptosis, which can lead to the occurrence of cancer. TIPE1 is an enhancer of cell apoptosis, which can induce cell apoptosis and which has an anti-tumor effect. As a negative regulator of inflammation and immunity, TIPE2 can prevent the occurrence of some tumors [6]. Among these, TIPE3, identified in 2008, is the least studied member of the TIPE family [7]. TIPE3 is highly expressed in most human tumor cell lines, such as lung cancer cell line NCI-H727, bladder cancer cell line T24, and colon adenocarcinoma cell line HT-29, but with a low expression in the gastric cancer cell line. At the same time, it is significantly up-regulated in lung cancer, esophagus cancer, cervical cancer, and colon cancer [8]; it was found that TIPE3 overexpression significantly increased the migration, invasion, and proliferation of MCF-7 and MDA-MB-231 tumor cells in human breast cancer cell lines. On the contrary, knockdown of TIPE3 in MDA-MB-231 cells can significantly reduce the migration, invasion, and proliferation of tumor cells [9]. In human non-small-cell lung cancer (NSCLC), some researchers have found that the expression of TIPE3 on the plasma membrane is positively correlated with the T stage of NSCLC, indicating that TIPE3 located in the plasma membrane may play a role in promoting tumor development, whereas cytoplasmic TIPE3 may exert a negative effect [10]. Meanwhile, in human glioblastoma (GBM), overexpressed TIPE3 inhibits p38 phosphorylation and blocks p38 nuclear translocation, leading to the negative regulation of the p38 MAPK pathway and resulting in GBM cell survival [11]. These results suggest that the role of TIPE3 in different tumors is heterogeneous.

In this work, we mainly used clinical/surgical specimens, focusing on the correlation between the TIPE3 expression and tumor-immune infiltration and the prognosis of CRC patients. The expression level of TIPE3 in the cancer and adjacent tissues of 110 CRC patients was detected by immunohistochemistry (IHC). According to the score statistics of the IHC results, combined with the patients’ prognosis information, the survival curve of the TIPE3 expression in CRC tissues and the prognosis of patients were determined, and the correlation between the TIPE3 expression and prognosis was analyzed. At the same time, we investigated the relationship between the TIPE3 expression level and tumor immune invasion: IHC was used to detect the positive cell numbers of CD8+ T cells, CD20+ B cells, and CD66b+ neutrophils in the cancer and adjacent tissues of 110 CRC patients. Combined with the prognosis information of the patients, a corresponding survival curve was drawn, and we found that patients with a high TIPE3 expression had a lower survival rate, while patients with a low TIPE3 expression had a higher survival rate.

## Materials and methods

### Patients and specimens

Formalin-fixed paraffin-embedded (FFPE) samples from CRC patients (*n* = 227) were used in this study. All samples, including CRC tissue specimens and the corresponding adjacent normal mucosal tissue specimens (colorectal mucosal tissue more than 5 cm from the edge of the tumor tissue), were obtained from patients with primary histologically confirmed CRC between June 2015 to June 2016 at the First Affiliated Hospital of Anhui Medical University, Hefei, China. The inclusion criteria were: 1) patients who underwent radical colorectal cancer surgery; 2) patients with available follow-up data and clinicopathological characteristics; 3) patients with no history of cancer treatment, without other malignant tumors; and 4) patients without serious perioperative complications or death. Informed consent was obtained from all participants before the study, which was approved by the Ethics Committee of the First Affiliated Hospital of Anhui Medical University (NO: LLSC2021010).

### Follow-up

All patients were followed up on a long-term basis until June 31, 2021, by phone call, outpatient follow-up, and inpatient hospital review. The follow-up period ranged from 1 to 67 months. The details are listed in Table 1.

**Table 1:** The Correlation Between TIPE3 Expression and Clinicopathological Features in CRC Patients

Characteristics	TIPE3 expression in Tumor tissues		*p*-value	TIPE3 expression in Adjacent normal tissues		*p*-value
	Low(*n*=34)	High(*n*=76)		Low(*n*=72)	High(*n*=38)	
Gender						
Male	24 (70.6)	46 (60.5)	0.424	49 (68.1)	21 (55.3)	0.264
Fmale	10 (29.4)	30 (39.5)		23 (31.9)	17 (44.7)	
Lymph node metastasis						
Negative	17 (50.0)	42 (55.3)	0.761	40 (55.6)	19 (50.0)	0.723
Postive	17 (50.0)	34 (44.7)		32 (44.4)	19 (50.0)	
Invasion						
I	1 ( 2.9)	0 ( 0.0)	0.489	1 ( 1.4)	0 ( 0.0)	0.491
II	4 (11.8)	11 (14.5)		12 (16.7)	3 ( 7.9)	
III	23 (67.6)	50 (65.8)		45 (62.5)	28 (73.7)	
IV	6 (17.6)	15 (19.7)		14 (19.4)	7 (18.4)	
TNM						
T1	5 (14.7)	11 (14.5)	0.851	13 (18.1)	3 ( 7.9)	0.356
T2	12 (35.3)	31 (40.8)		27 (37.5)	16 (42.1)	
T3	17 (50.0)	34 (44.7)		32 (44.4)	19 (50.0)	
Grade						
Low	4 (11.8)	17 (22.4)	0.395	13 (18.1)	8 (21.1)	0.727
Moderate	29 (85.3)	56 (73.7)		57 (79.2)	28 (73.7)	
High	29 (85.3)	56 (73.7)		2 ( 2.8)	2 ( 5.3)	
Location (%)						
Location (%)	29 (85.3)	56 (73.7)	0.554	28 (38.9)	17 (44.7)	0.697
Colon	22 (64.7)	43 (56.6)		44 (61.1)	21 (55.3)	
Status (%)						
Survive	29 (85.3)	42 (55.3)	0.005**	56 (77.8)	15 (39.5)	<0.001
Dead	5 (14.7)	34 (44.7)		16 (22.2)	23 (60.5)	
CEA	5.61 [2.96, 20.33]	3.70 [2.31, 7.77]	0.078	3.80 [2.36, 7.93]	4.96 [2.53, 14.77]	0.343
CA-199	13.71 [9.12, 32.54]	10.64 [6.11, 26.79]	0.196	11.34 [6.91, 21.13]	13.61 [6.26, 37.21]	0.418
Age	65.50 [57.25, 77.75]	62.00 [53.75, 68.00]	0.179	63.00 [56.50, 72.00]	62.00 [53.25, 70.00]	0.792
Survive Time	64.00 [63.00, 64.75]	63.00 [35.50, 64.00]	0.006**	64.00 [63.00, 64.00]	56.00 [22.25, 63.00]	<0.001
WBC	6.32 [4.88, 6.88]	5.72 [4.64, 7.35]	0.874	5.74 [4.56, 7.04]	6.29 [4.91, 7.26]	0.622
Neutrophil	3.60 [2.95, 4.69]	3.23 [2.75, 4.76]	0.514	3.48 [2.80, 4.77]	3.29 [2.80, 4.73]	0.664
Lymphocyte	1.46 [1.23, 1.87]	1.48 [1.12, 1.95]	0.828	1.40 [1.08, 1.77]	1.66 [1.29, 2.17]	0.011*
Monocyte	0.37 [0.30, 0.45]	0.36 [0.28, 0.48]	0.864	0.36 [0.28, 0.45]	0.38 [0.28, 0.55]	0.196
Hb	131.00 [113.25, 137.00]	125.50 [97.75, 132.25]	0.113	127.50 [107.25, 134.00]	122.50 [101.75, 131.50]	0.336
PLT	214.50 [171.50, 280.00]	217.00 [171.00, 265.25]	0.910	1218.00 [169.50, 266.25]	196.00 [173.00, 270.50]	0.797

*CEA* Carcinoembryonic antigen, *CA-199* Carbohydrate antigen 199, *WBC* White blood cell, *Hb* Hemoglobin, *PLT* Platelet

*p < 0.05, **p < 0.01

### Endpoint of study

The endpoint of this study was the overall survival (OS). OS was defined as the time from radical CRC surgery to death or the last follow-up for surviving patients.

### Immunohistochemistry

Based on the findings of previous studies [12, 13], we selected three prognostic immune biomarkers for IHC staining: cytotoxic T cells (CD8), B cells (CD20), and neutrophils (CD66b) [14]. All FFPE samples were cut into 4-μm sections (Leica, Germany) and then processed for the IHC assay, as previously described [15]. Following incubation with an antibody against human TIPE3 (Boster, A14951, Wuhan, China), CD8 (Affinity, AF5126, Changzhou, China), CD20 (Affinity, DF13319, Changzhou, China), and CD66b (Affinity, DF10151, Changzhou, China), the sections were stained using the Envision System (PV6001, ZSGB-BIO, China). The IHC results were evaluated by two independent observers blinded to the clinical outcome. First, the general condition of the tissue section was assessed under an optical microscope (Olympus Upright Microscope BX53, Olympus, Japan) at a low power field (100x). Then, five representative high-power fields (200x) of each tissue section were photographed and scored. Then, the average of five fields was taken as the positive cell score.

### Immunohistochemical score

We used different scoring systems for different proteins; for the TIPE3 expression, a combined immunohistochemical score was calculated using the percentage of positively stained cells and signal intensity; the percentage of TIPE3-positive cells was graded as follows: 0, <1%; 1, 1–20%; 2, 21–40%; 3, 41–60%; 4, 61%–80%; 5, ≥81%, and the signal intensity, as follows: 0, no staining; 1, weak staining; 2, moderate staining; 3, strong staining. Then, the individual scores were added to obtain the combined score. For immune cells, the number of positive cells was the score for this field.

### Clinicopathological parameters

The basic information of each patient was recorded in this study (Table 1), including gender; age; tumor location; tumor grade; preoperative carcinoembryonic antigen (CEA) level; preoperative carbohydrate antigen 199 (CA199) level; tumor invasion depth; lymph node metastasis status; survival time; and TNM Classification of Malignant Tumors (TNM) stage. Patients with TNM staging were restaged according to the 7th edition of the American Joint Committee on Cancer (AJCC) tumor staging manual. CEA and CA199 levels above 5 ng/mL and 27 µg/mL, respectively, were considered abnormal. The relevant experiments were approved by the Ethics Committee of the First Affiliated Hospital of Anhui Medical University (NO: LLSC2021010).

### Statistical analysis

Data were analyzed using the SPSS software (version 22.0; IBM Corporation) with default parameters and the R software (version 4.1.0). Continuous variables were expressed as the median (interquartile range [IQR]) or mean (standard deviation [SD]), as appropriate. Frequencies and percentages were used to express categorical variables.

Pearson’s χ2 or Fisher’s exact test was conducted to analyze categorical variables, and the Student’s t-test or rank-sum test was used to examine continuous variables. Univariate and multivariate analyses were performed using the Cox proportional risk regression analysis to screen potential prognostic factors for OS. We determined the optimal cutoff values of the TIPE3, CD8, CD20, and CD66b expressions using survminer from the R package (version 0.4.9). Subsequently, a Kaplan–Meier plot and log-rank test were applied in terms of the high- and low-expression TIPE3, CD8, CD20, and CD66b groups (expressed as total survival time [OS]). A *p*-value of <0.05 was considered statistically significant.

## Results

### Clinical characteristics

**Fig. 1:**
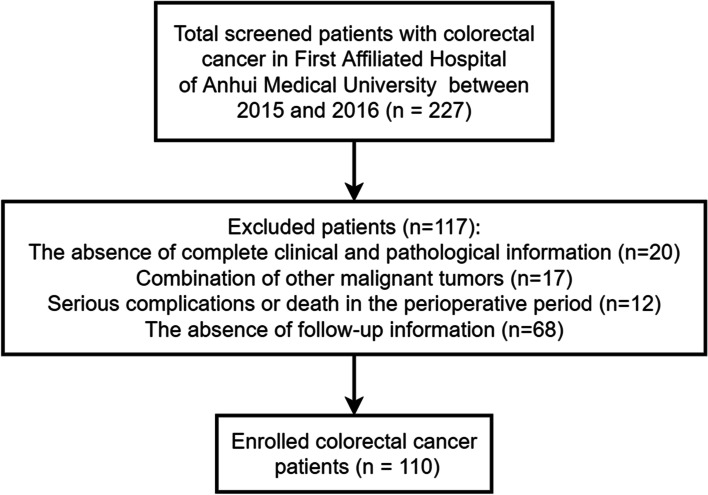
Flow chart of the study population

The pathological information of the enrolled CRC patients is presented in Table 1. FFPE samples from CRC patients (*n* = 227) were collected in this study. Of these, 117 cases were excluded and 110 were eventually enrolled. The patient exclusion criteria were: 1) the absence of complete clinical and pathological information (*n* = 20), 2) a combination of other malignant tumors (*n* = 17), 3) serious complications or death in the perioperative period (*n* = 12), and 4) the absence of follow-up information (*n* = 68). In total, 110 patients were finally enrolled, with a median age of 62.0 years old (interquartile range: 54.0–71.0 years old), including 70 males and 40 females. The flow chart of the study population is shown in Fig. 1.

### TIPE3 expression in CRC tissues

**Fig. 2:**
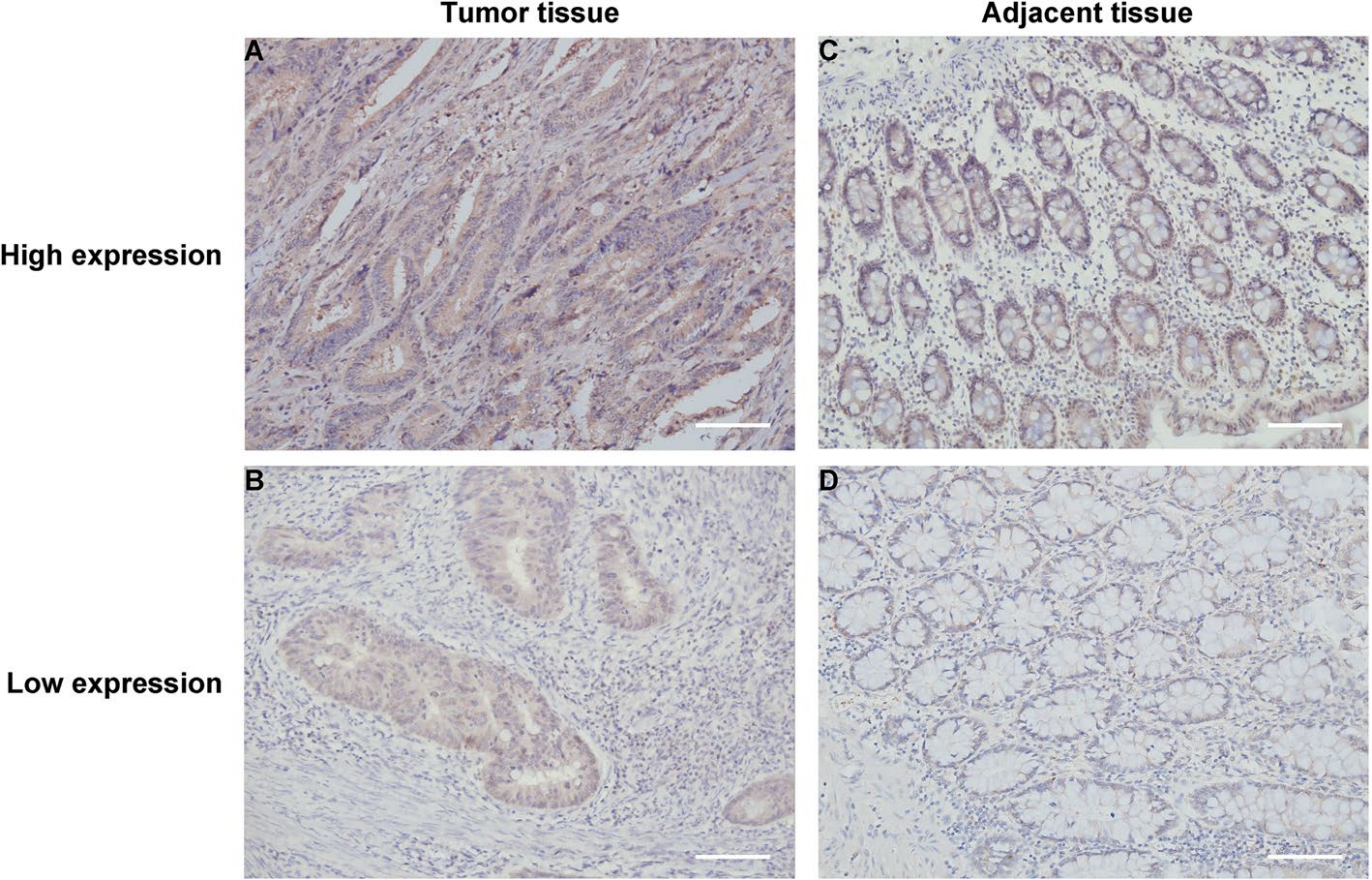
Images of immunohistochemical staining of TIPE3 in CRC Tumor tissue and Adjacent tissue (200×): **A**, **B** Tumor tissue: **A** TIPE3 high expression; **B** TIPE3 low expression; **C**, **D** Adjacent tissue: **C** TIPE3 high expression; **D** TIPE3 low expression. CRC, colorectal cancer; Scale bar = 100 μm

First, we performed IHC staining of TIPE3 in the tumor tissues and adjacent normal tissues of 110 CRC specimens and found varying levels of the TIPE3 expression in both tissue types, with a high expression in the cytoplasm and low levels in the nucleus (Fig. 2). After IHC scoring, the median score was taken as the cutoff value, and the cancer tissues were divided into a low TIPE3 expression group (≤4.9; *n* = 34) and a high TIPE3 expression group (>4.9; *n* = 76). Similarly, the adjacent normal tissues were divided into a low TIPE3 expression group (≤3.4; *n* = 72) and a high TIPE3 expression group (>3.4; *n* = 38). A statistical analysis showed that 69.10% (76/110) and 30.90% (34/110) of cases exhibited high and low TIPE3 expression levels in cancer tissues, respectively, while 34.55% (38/110) and 65.45% (72/110) of cases exhibited high and low TIPE3 expressions in adjacent normal tissues, respectively. Overall, the expression of TIPE3 in cancer tissues was significantly higher than in adjacent normal tissues.

### Correlation between TIPE3 expression and clinicopathological features in CRC patients

During the correlation analysis between the TIPE3 expression and clinicopathological factors (Table 1), the TIPE3 expression in tumor tissues was significantly correlated with survival status (*p* = 0.005) and survival time (*p* = 0.006). Similarly, the TIPE3 expression in the adjacent normal tissues correlated with survival status (*p*<0.001) and survival time (*p*<0.001). Interestingly, there was also a significant correlation between the TIPE3 expression and the blood lymphocyte count (*p* = 0.011). However, no differences in the TIPE3 expression were found after stratifying by gender, age, TNM stage, depth of tumor invasion, and tumor grade.

### Correlation between TIPE3 expression and tumor-immune infiltration

**Fig. 3:**
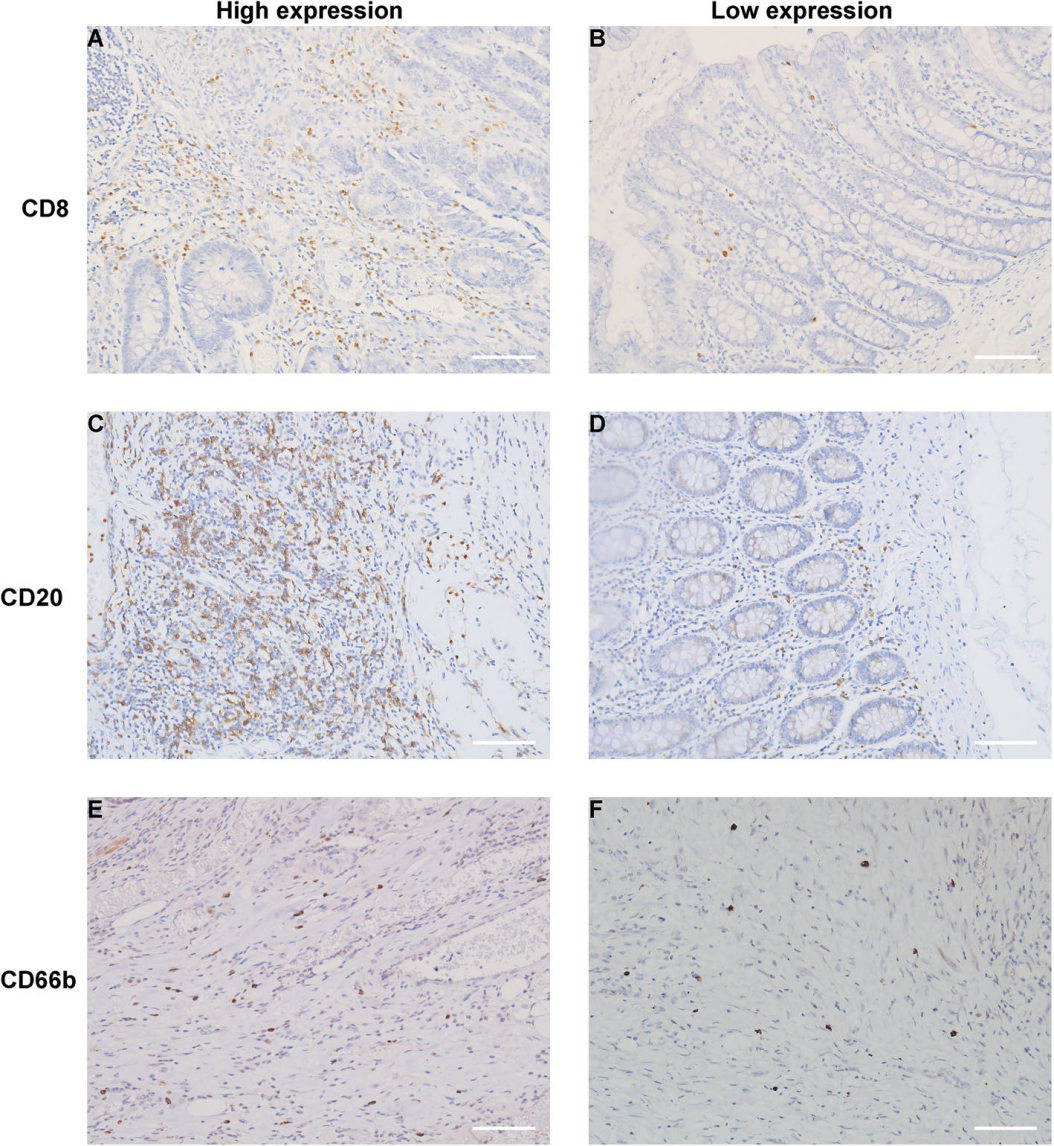
Images of immunohistochemical staining immune cells in CRC tissue (200×): **A**, **B** Immunohistochemical staining of CD8+ T cells: **A** High expression; **B** Low expression; **C**, **D** Immunohistochemical staining of CD20+ B cells: **C** High expression; **D** Low expression; **E**, **F** Immunohistochemical staining of CD66b+ neutrophils: **E** High expression; **F** Low expression. Scale bar = 100 μm

We performed IHC staining of CD8+ T cells, CD20+ B cells, and CD66b+ neutrophils in the tumor tissues and adjacent normal tissues of the 110 CRC specimens and observed the staining results (Fig. 3). The TIPE3 expression and the number of immune cells were scored (Table 2), and the results showed that TIPE3 was significantly correlated with the number of CD20+ B cells in tumor tissues (*p* = 0.004), while no significant results were observed for the other markers. Taken together, our results suggest the TIPE3 expression may be associated with tumor-immune infiltration.

**Table 2:** Correlation Between TIPE3 Expression and tumor immune infiltration

Characteristics	TIPE3 expression in Tumor tissues		*p*-value	TIPE3 expression in adjacent normal tissues		*p*-value
	Low(*n*=34)	High(*n*=76)		Low(*n*=72)	High(*n*=38)	
CD8_T	150.00 [80.00, 285.00]	100.00 [60.00, 200.00]	0.296	150.00 [70.00, 265.00]	100.00 [50.00, 147.50]	0.060
CD8_A	80.00 [40.00, 172.50]	80.00 [40.00, 175.00]	0.825	80.00 [40.00, 162.50]	80.00 [42.50, 200.00]	0.902
CD20_T	234.00 [145.00, 264.25]	190.00 [112.00, 267.00]	0.259	235.50 [141.75, 275.00]	151.50[71.75, 240.50]	0.004**
CD20_A	94.50 [28.50, 180.50]	91.50 [38.75, 175.50]	0.948	86.00 [26.75, 176.50]	98.00 [47.75, 174.50]	0.432
CD66b_T	11.80 [8.40, 19.00]	18.34 [9.00, 63.25]	0.052	14.34 [8.19, 27.50]	17.34 [10.54, 94.00]	0.170
CD66b_A	18.55 [11.32, 27.00]	19.25 [11.48, 46.00]	0.520	18.25 [11.11, 35.00]	20.60 [13.18, 48.00]	0.201

*CD8* CD8+ T cells, *CD20* CD20+ B cells, *CD66b* CD66b+ neutrophils, *T* Tumor tissues, *A* Adjacent normal tissues

**p <0.01

### Correlation between TIPE3 expression and prognosis

**Fig. 4:**
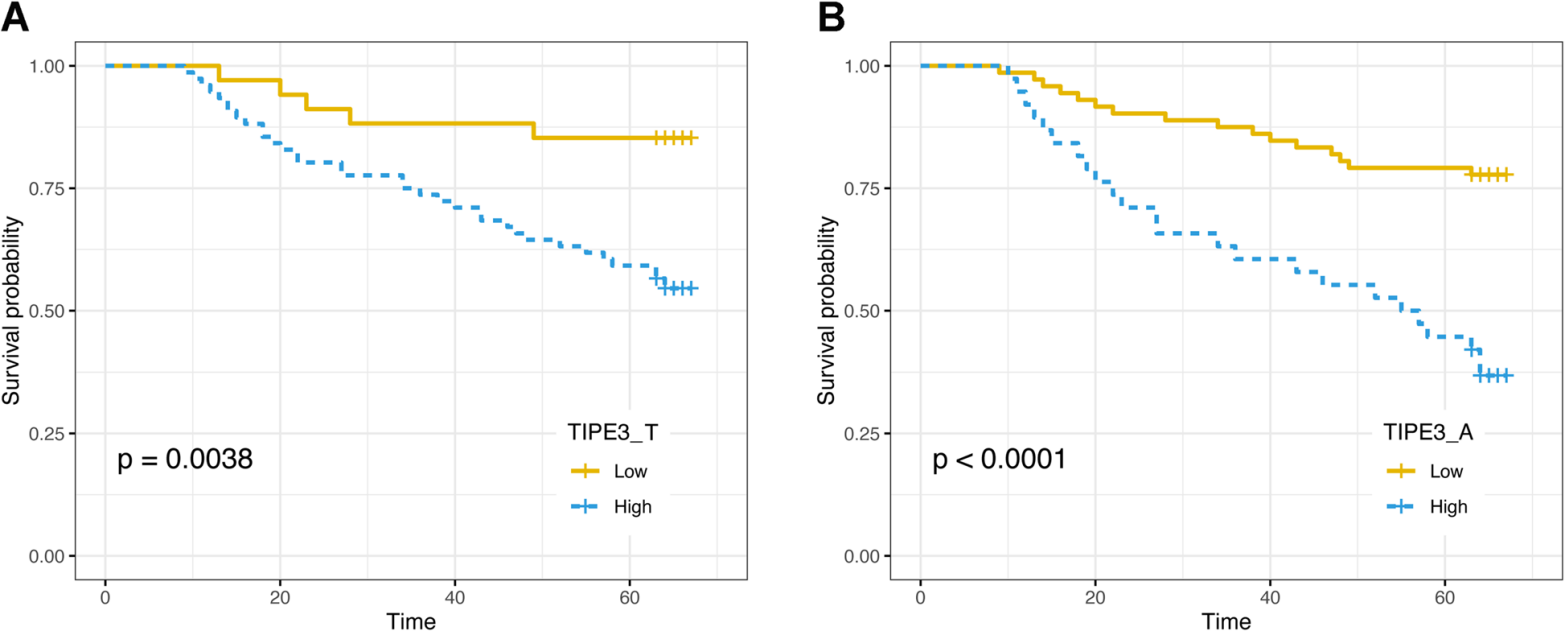
Kaplan-Meier survival curves associated with TIPE3 expression of CRC patients: **A** Survival curve of tumor tissues for CRC patients; **B** Survival curve of adjacent normal tissues for CRC patients. T, tumor tissues; A, adjacent normal tissues

After long-term follow-up, mortality was observed in 35.35% (*n* = 39) of CRC patients. Then, based on the prognostic information, we conducted a Kaplan–Meier survival analysis based on the TIPE3 expression (Fig. 4), which was significantly correlated with the survival rate of patients with tumor tissues (*p* = 0.0038) and adjacent normal tissues (*p*<0.0001); a high TIPE3 expression correlated with a lower survival rate.

Finally, we conducted a Cox regression analysis of univariate variables and multivariate variables (Table 3), and a univariate regression analysis showed that the TIPE3 expression in tumor tissues (HR = 3.647, 95% CI: 1.425–9.332, *p* = 0.007), the TIPE3 expression in adjacent normal tissues (HR = 3.557, 95% CI: 1.884–6.781, *p*<0.001), the number of CD8+ T cells in tumor tissues (HR = 0.461, 95% CI: 0.240–0.889, *p* = 0.020), the number of CD20+ B cells in tumor tissues (HR = 0.468, 95% CI: 0.243–0.900, *p* = 0.023), the number of CD20+ B cells in adjacent normal tissues (HR = 0.496, 95% CI: 0.258–0.955, *p*=0.023), the number of CD66b+ neutrophils in tumor tissues (HR = 2.696, 95% CI: 1.340–5.424, *p* = 0.005), the number of CD66b+ neutrophils in adjacent normal tissues (HR = 3.359, 95% CI: 1.634–6.904, *p*<0.001), lymphatic metastasis (HR = 2.358, 95% CI: 1.224–4.543, *p* = 0.010), TNM stage (HR = 1.924, 95% CI: 1.15–3.218, *p* = 0.013), and tumor grade (HR = 0.476, 95% CI: 0.246–0.921, *p* = 0.027) were significantly correlated with OS. These prognostic factors were then subjected to multivariate regression analysis, and the results showed that the TIPE3 expression in adjacent normal tissues (HR = 3.215, 95% CI: 1.569–6.586, *p*=0.0014), the number of CD8+ T cells in adjacent normal tissues (HR = 0.387, 95% CI: 0.184–0.813, *p* = 0.0122), and the number of CD66b+ neutrophils in adjacent normal tissues (HR = 2.474, 95% CI: 1.036–5.905, *p* = 0.0413) were prognostic factors affecting the OS rate of CRC patients.

**Table 3:** Univariate and multivariate cox proportional hazards models for the predictors of overall survival

**Variable**	**Univariable**			**Multivariable**		
	**HR**	**95% CI**	***p*-value**	**HR**	**95% CI**	***p*-value**
TIPE3_T	3.647	(1.425 – 9.332)	0.007**	2.395	(0.896-6.399)	0.0816
TIPE3_A	3.577	(1.884 – 6.791)	<0.001	3.215	(1.569-6.586)	0.0014**
CD8_T	0.461	(0.240 - 0.889)	0.020*	0.635	(0.314-1.284)	0.2062
CD8_A	0.542	(0.288 - 1.018)	0.057	0.387	(0.184-0.813)	0.0122*
CD20_T	0.468	(0.243 - 0.900)	0.023*	0.701	(0.329-1.493)	0.3568
CD20_A	0.496	(0.258 – 0.955)	0.036*	0.558	(0.276-1.128)	0.1043
CD66b_T	2.696	(1.340 – 5.424)	0.005**	0.965	(0.385-2.423)	0.9401
CD66b_A	3.359	(1.634 – 6.904)	<0.001	2.474	(1.036-5.905)	0.0413*
Gender	1.438	(0.764 - 2.709)	0.261			
Age	0.985	(0.964 - 1.007)	0.188			
Invasion	1.638	(0.962 - 2.791)	0.069	0.972	(0.417-2.263)	0.9471
Grade	0.476	(0.246 - 0.921)	0.027*	0.620	(0.287-1.338)	0.2234
TNM	1.924	(1.150 - 3.218)	0.013*	1.076	(0.214-5.410)	0.9292
Lymph node metastasis	2.358	(1.224 - 4.543)	0.010*	1.553	(0.213-11.310)	0.6637
Hb	0.999	(0.987 - 1.012)	0.928			
WBC	0.978	(0.832 - 1.150)	0.790			
PLT	1.001	(0.997 - 1.005)	0.674			
Neutrophil	0.967	(0.817 - 1.145)	0.698			
Monocyte	0.805	(0.109 - 5.971)	0.832			
Lymphocyte	1.132	(0.663 - 1.932)	0.651			

*HR* Hazard ratio, *T* Tumor tissues, *A* Adjacent normal tissues, *Hb* Hemoglobin, *WBC* White blood cell, *PLT* Platelet

*p <0.05, **p <0.01

## Discussion

The most effective form of prevention and early treatment of CRC is colonoscopy, which can detect precancerous lesions and early cancers to improve survival and prognosis rates significantly [16]. Current evidence suggests that through early screening, diagnosis, and treatment, patients with rectal cancer have a better prognosis, with a five-year survival rate of about 90% and a metastasis rate of only 14% [17]. In recent years, researchers have achieved significant strides in bringing awareness to the importance of early screening [18], made improvements to surgical techniques [19], and developed targeted therapy [20], which have significantly improved the survival rate of CRC patients. In our work, we found that patients with a high TIPE3 expression had a lower survival rate, while patients with a low TIPE3 expression had a higher survival rate. However, the type of TIPE positive cells was not analyzed in detail. Univariate regression analysis showed that the expression of TIPE3 and the number of CD66b+ neutrophils were risk factors for the OS of CRC patients; and the number of CD8+ T cells, the number of CD20+ B cells, and tumor grade were protective factors for the OS of CRC patients. These prognostic factors were then subjected to multivariate regression analysis, and the results show that the expression of TIPE3, the number of CD8+ T cells, and the number of CD66b+ neutrophils are prognostic factors affecting the OS rate of CRC patients, and TIPE3 is correlated with survival rate.

Our study found that the TIPE3 expression in CRC cancer tissues was significantly higher than in adjacent normal tissues, which may be related to the fact that TIPE3 can promote the occurrence and development of tumors. It is widely thought that at the cancerous site, TIPE3 is stimulated by the tumor environment, resulting in a higher TIPE3 expression, which in turn accelerates the transport rates of PIP2 and PIP3, two lipid second messengers; this activates the PI3K/AKT and MEK-ERK signaling pathways, which further promotes the growth, proliferation, and migration of tumor cells, accounting for the malignancy and poor prognosis. Combined with the prognostic information of CRC patients, it is suggested that a high TIPE3 expression predicts a poor prognosis, and the survival rate of these patients is significantly lower than that of patients with a low TIPE3 expression, consistent with the results of the TCGA database analysis (Supplementary Fig. 1). We noted that Zhong’s analysis of 49 CRC samples showed no correlation between TIPE3 mRNA expression levels and patients’ clinicopathological features, while the TIPE3 protein expression level in the tumor group was lower than that in the adjacent group [21]. This contrasts our current results, possibly because of the different number of samples tested or the existence of tumor heterogeneity. In our work, 110 samples were used, and IHC staining was performed to count the number of TIPE3-positive cells in the tumor and adjacent tissues, and two conditions—high and low TIPE3 expressions—in tumor tissues were found, among which the number of high-expression samples was 76 and the number of low-expression samples was 34. This result is consistent with most studies [22, 23].

Tumor-infiltrating immune cells are an important component of tumor tissue and play a crucial role in tumor occurrence and development. T cells are key tumor-infiltrating immune cells [24], and CD8+ T cells, a T-cell subtype, participate in the immune surveillance of tumor antigens during tumor development. T cell antigen receptors activate CD8+ T cells, rapidly proliferate and differentiate into cytotoxic T lymphocytes, and finally eliminate cancer cells through cellular immunity. Ample literature suggests that the extensive infiltration of CD8+ T cells in tumors is associated with good patient prognosis [25–28]. In our work, we found that the expression of TIPE3 and the number of CD8+ T cells were prognostic factors affecting the OS rate of CRC patients. Although our study did not find a significant correlation between the TIPE3 expression and the number of tumor-infiltrating immune cells, a Cox regression analysis showed that the TIPE3 expression and CD8+ T cells jointly affect patient survival (Table 3 and Supplementary Fig. 2), suggesting they have significant prospects for clinical applications as prognostic indicators.

## Conclusion

Using clinical samples from 110 CRC patients, we found the TIPE3 protein to be upregulated in CRC cancer tissues and to be significantly correlated with patients’ OS rate. The high TIPE3 expression correlated with poor overall survival of the patients.

## Supplementary Information

**Additional file 1: Supplementary Figure 1.** Overall survival curves associated with TIPE3 expression in the TCGA database. **Supplementary Figure 2.** Kaplan-Meier survival curves associated with immune cells of CRC patients: (A, B) Survival curves associated with CD8+ T cells of CRC patients: (A) Survival curve of tumor tissues; (B) Survival curve of adjacent normal tissue; (C, D) Survival curves associated with CD20+ B cells of CRC patients: (C) Survival curve of tumor tissues; (D) Survival curve of adjacent normal tissue; (E, F) Survival curves associated with CD66b+ neutrophils of CRC patients: (E) Survival curve of tumor tissues; (F) Survival curve of adjacent normal tissue. T, tumor tissues; A, adjacent normal tissue.

## Data Availability

The data that support the findings of this study are available from the corresponding authors but restrictions apply to the availability of these data, which were used under license for the current study, and so are not publicly available. Data are however available from the authors upon reasonable request and with permission of Anhui Medical University and The First Affiliated Hospital of Anhui Medical University.

## Abbreviations

AJCC: American Joint Committee on Cancer
B cells: B- T-lymphocyte
CA199: Carbohydrate antigen 199
CD: Cluster of Differentiation
CEA: Carcinoembryonic antigen
CI: Confidence interval
CRC: Colorectal cancer
DED: Death effector domain
FFPE: Formalin-fixed paraffin-embedded
HR: Hazard ratio
IHC: Immunohistochemistry
IQR: interquartile range
OS: Overall survival
PIP2: Phosphatidylinositol 4,5-bisphosphate
PIP3: Phosphatidylinositol 3,4,5-trisphosphate
SD: Standard deviation
T cells: T-lymphocyte
TNFAIP8L3/TIPE3: Tumor necrosis factor α-induced protein 8 like 3
