# Supplementary material for: Tumor necrosis factor-α-inducible protein 8-like protein 3 (TIPE3): a novel prognostic factor in colorectal cancer
Source: BMC Cancer. 2023 Feb 8;23:131. doi: 10.1186/s12885-023-10590-2 (PMC9909977; doi:10.1186/s12885-023-10590-2)
Supplement: Supplementary file 1 — Additional file 1: Supplementary Figure 1. Overall survival curves associated with TIPE3 expression in the TCGA database. Supplementary Figure 2. Kaplan-Meier survival curves associated with immune cells of CRC patients: (A, B) Survival curves associated with CD8+ T cells of CRC patients: (A) Survival curve of tumor tissues; (B) Survival curve of adjacent normal tissue; (C, D) Survival curves associated with CD20+ B cells of CRC patients: (C) Survival curve of tumor tissues; (D) Survival curve of adjacent normal tissue; (E, F) Survival curves associated with CD66b+ neutrophils of CRC patients: (E) Survival curve of tumor tissues; (F) Survival curve of adjacent normal tissue. T, tumor tissues; A, adjacent normal tissue. [file 12885_2023_10590_MOESM1_ESM.doc]

**Tumor necrosis factor-α-inducible protein 8-like protein 3 (TIPE3): A novel prognostic factor in colorectal cancer**

Yue Xu^#1^, Yong Zhu^#2,3^, Hengbo Xia^#4^, Yanan Wang^1^, Lin Li^1^, Hong Wan^2,3^, Shuping Zhang^2,3^, Aman Xu^2,3,4^, Liecheng Wang^1^, Jiao Gong*^4^ and Pingping Zhang*^1^

1. Department of Physiology, School of Basic Medical Sciences, Anhui Medical University, Hefei, China;

2.The First Affiliated Hospital of Anhui Medical University，Hefei, China;

3. Anhui Public Health Clinical Center, Hefei, China;

4. Department of General Surgery, First Affiliated Hospital of Anhui Medical University, Hefei, China;

5. Department of Laboratory Medicine, Third Affiliated Hospital of Sun Yat-sen University, Guangzhou, China.

^#^These authors contributed equally: Yue Xu, Yong Zhu, Heng B. Xia.

*Correspondence: Jiao Gong, [gongjiao@mail2.sysu.edu.cn](mailto:gongjiao@mail2.sysu.edu.cn); Tel: +86-18666018674;

Pingping Zhang, [791663310@qq.com](mailto:791663310@qq.com); Tel: +86-551-65161132.

**
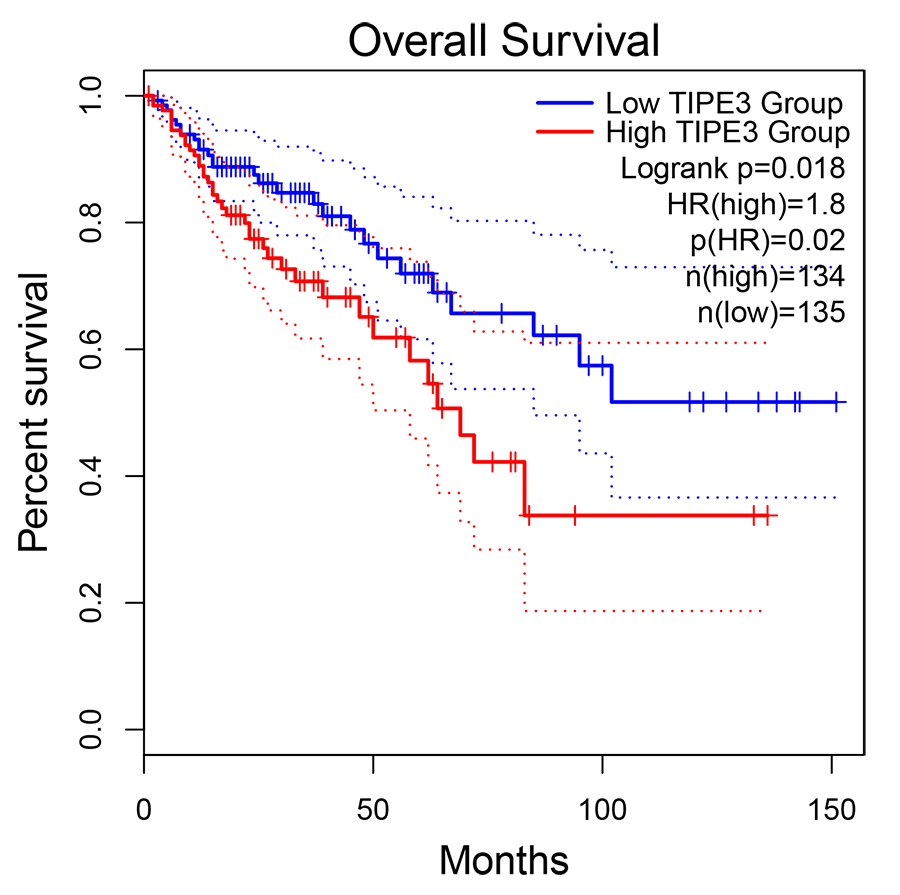
**

**Supplementary Figure 1 Overall survival curves associated with TIPE3 expression in the TCGA database**

**
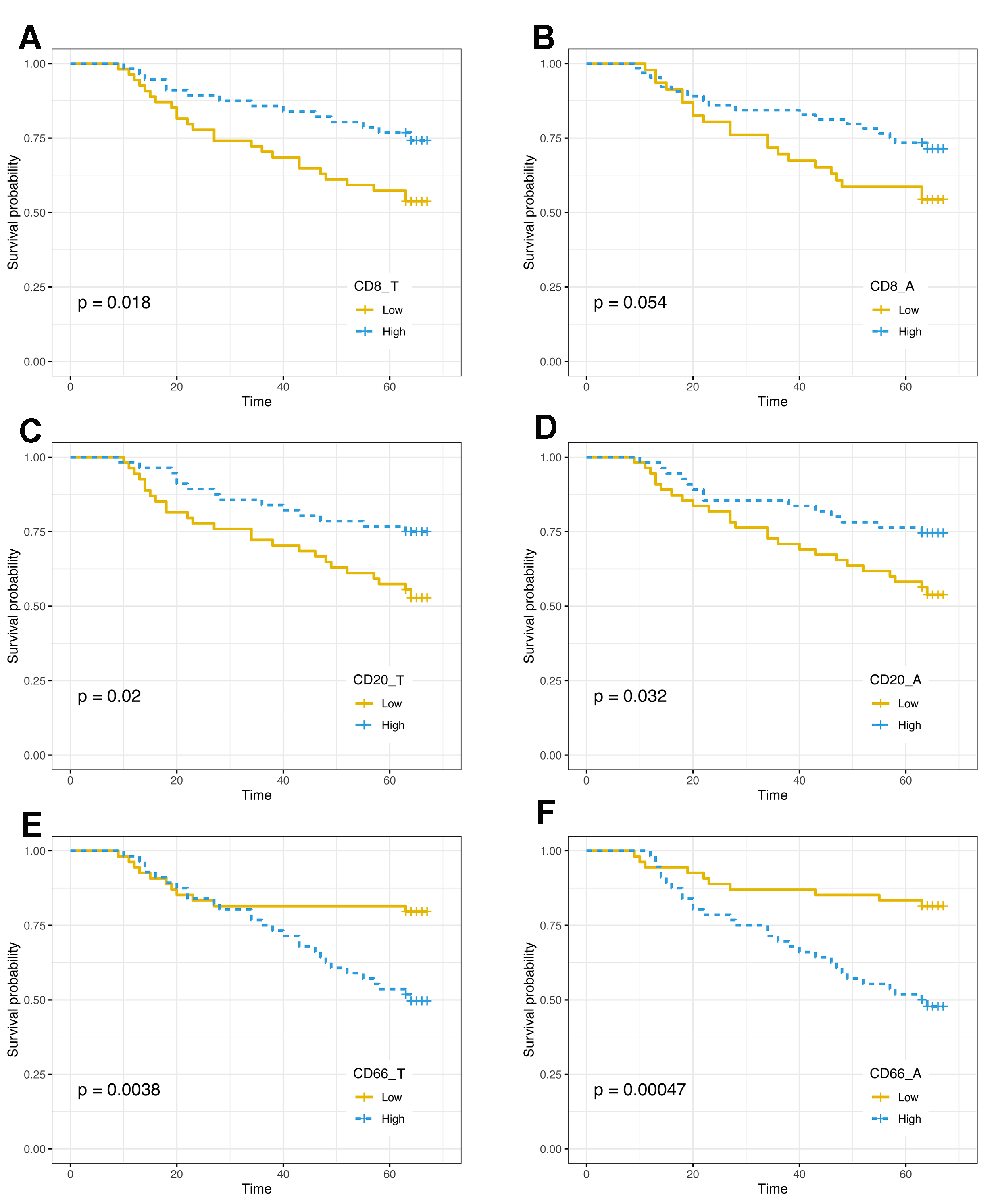
**

**Supplementary Figure 2 Kaplan-Meier survival curves associated with immune cells of CRC patients: (A, B)** Survival curves associated with CD8+ T cells of CRC patients: (A) Survival curve of tumor tissues; **(B)** Survival curve of adjacent normal tissue; **(C, D)** Survival curves associated with CD20+ B cells of CRC patients: **(C)** Survival curve of tumor tissues; **(D)** Survival curve of adjacent normal tissue**; (E, F)** Survival curves associated with CD66b+ neutrophils of CRC patients: **(E)** Survival curve of tumor tissues; **(F)** Survival curve of adjacent normal tissue**.** T, tumor tissues; A, adjacent normal tissue.
